# Verification of extraoral versus intraoral scanning techniques: fit accuracy implications for 3D printed and milled zirconia crowns (An in-vitro study)

**DOI:** 10.1186/s12903-025-07577-9

**Published:** 2026-01-17

**Authors:** Rogina M. Hassan, Yomna Ibrahim, Rewaa G. AboELHassan, Amir Shoukry Azer

**Affiliations:** 1 Conservative Dentistry Department, Faculty of Dentistry, Alexandria University, Alexandria, Egypt; 2 Dental Biomaterials Department, Faculty of Dentistry, Alexandria University, Alexandria, Egypt

**Keywords:** 3D printing, Extraoral scanners, Internal fit, Intraoral scanners, Marginal fit

## Abstract

**Background:**

Marginal and internal fit significantly influence the longevity of fixed restorations. This study aims to evaluate the accuracy of extraoral and intraoral scanning techniques for assessing gaps of 3D printed and milled zirconia crowns.

**Methods:**

Milled (*n* = 12) and 3D printed zirconia crowns (*n* = 12) were fabricated. Triple scan protocol was implemented for each specimen in both groups using extraoral and intraoral scanners to determine marginal and internal fit. The marginal fit was compared to predetermined marginal gap identified using stereomicroscope, whereas the internal fit was assessed in relation to a reference standard of cement space set at 70 μm during the crown design process. Comparisons between different scanners were conducted using an independent t-test for internal fit and one-way ANOVA for marginal fit.

**Results:**

Superimposition scans obtained from extraoral scanner revealed significantly lower gap values both internally and marginally when compared to the intraoral scans for both milled (*P* < 0.001, *P* = 0.04) and printed (*P* < 0.001, *P* < 0.001) zirconia. The values from both methods showed significant differences in internal fit compared to the reference standard (*P* < 0.001). The extraoral scanner showed no significant difference in marginal fit values when compared to stereomicroscope.

**Conclusions:**

The type of scanner used and the fabrication technique both influenced the accuracy of marginal and internal fit measurements. This verification provides an insight into the accuracy of extraoral and intraoral scanners in the assessment of internal and marginal fit of monolithic zirconia restorations fabricated by milling and 3D printing to aid in improving restoration retention and longevity of the fixed prostheses.

## Background

The advent of digital dentistry has revolutionized the way dental impressions are captured, leading to the development of intraoral and extraoral scanners. These technologies have become essential tools in prosthodontics, particularly for fabrication of zirconia crowns and for the evaluation of their marginal and internal fit [1].

Zirconia restorations are conventionally fabricated using subtractive milling. However, this method has several limitations, including challenges in milling thin margins without risking chipping and difficulties in producing restorations with intricate grooves and complex shapes [2–4]. Moreover, this approach generates significant material waste, leads to deterioration of the milling tools, and incurs high production costs [5].

The rapid advancement of materials and techniques in additive manufacturing (AM) has made it possible to produce monolithic zirconia restorations [6]. There are various technologies in AM of zirconia including, selective laser sintering (SLS), stereolithography (SLA) and digital light processing (DLP) [7]. DLP technique involves curing photosensitive slurries, where the polymer network serves as a binder for the ceramic particles. The resulting composites consist of separate ceramic particles bound together by the polymerized binder, creating the final green bodies. Excess or uncured material is then eliminated and components are sintered to attain their final properties [8, 9].

In single crown restorations, both marginal and internal fit are critical factors that significantly influence the longevity of the restoration [10]. The internal fit can be assessed in both axial and occlusal directions, which can impact crown retention and marginal fit [11, 12]. Holmes et al. [13] defined marginal gap as the vertical distance from the crown margin to the tooth’s finish line. Various methods have been utilized to evaluate the fit of crowns, including silicone replica technique (SRT), three-dimensional (3D) triple scanning, and direct-view methods [14–17].

The SRT is the most commonly used technique for assessing the fit of prosthetic restorations due to its effectiveness in estimating both marginal and internal discrepancies [18]. While it offers valuable insights into the fit of restorations, its limitations must be carefully considered to ensure accurate assessments. Digital scanners facilitate a more precise and detailed evaluation of the fit accuracy of dental restorations. By comparing the digital impression of the abutment with the digital scan of the intaglio surface of the crown using specialized software, a comprehensive view of the space between the abutment and crown is obtained [19].

The triple scan protocol, described by Holst et al. [20], has emerged as a reliable method for evaluating the fit accuracy of dental restorations. While both intraoral and extraoral scanners can be employed in this protocol, their performance may vary due to differences in accuracy, which can be influenced by several factors, including scanning technique, operator skill, environmental conditions, and scanner calibration [21].

Numerous studies have consistently demonstrated significant differences in accuracy between these two scanner types. Extraoral scanners generally provide higher accuracy for full arch and complex restorations, largely due to their advanced optical technologies and stable scanning environments. On the other hand, intraoral scanners have demonstrated commendable performance in scenarios requiring rapid assessment and partial arch captures, often producing results that are clinically acceptable for various applications. This distinction underscores the importance of selecting the appropriate scanning modality based on clinical requirements and the specific anatomical characteristics being captured [22].

Therefore, the main purpose of the present study was to verify the accuracy of extraoral versus intraoral scanners for fit assessment of 3D printed and milled zirconia crowns. The null hypothesis was that there is no significant difference in internal and marginal fit in scans acquired by extraoral and intraoral scanners.

## Methods

### Sample size Estimation

Sample size was based on 95% confidence level, 80% power, and effect size (d = 1.31) calculated based on marginal gap values in µm in the reference study by Refaie et al. to be 11 per group, increased to 12 to make up for laboratory processing errors. Sample size was calculated using MedCalc Statistical Software (version 19.0.5, Belgium) [23].

### Zirconia crowns fabrication

**Fig. 1:**
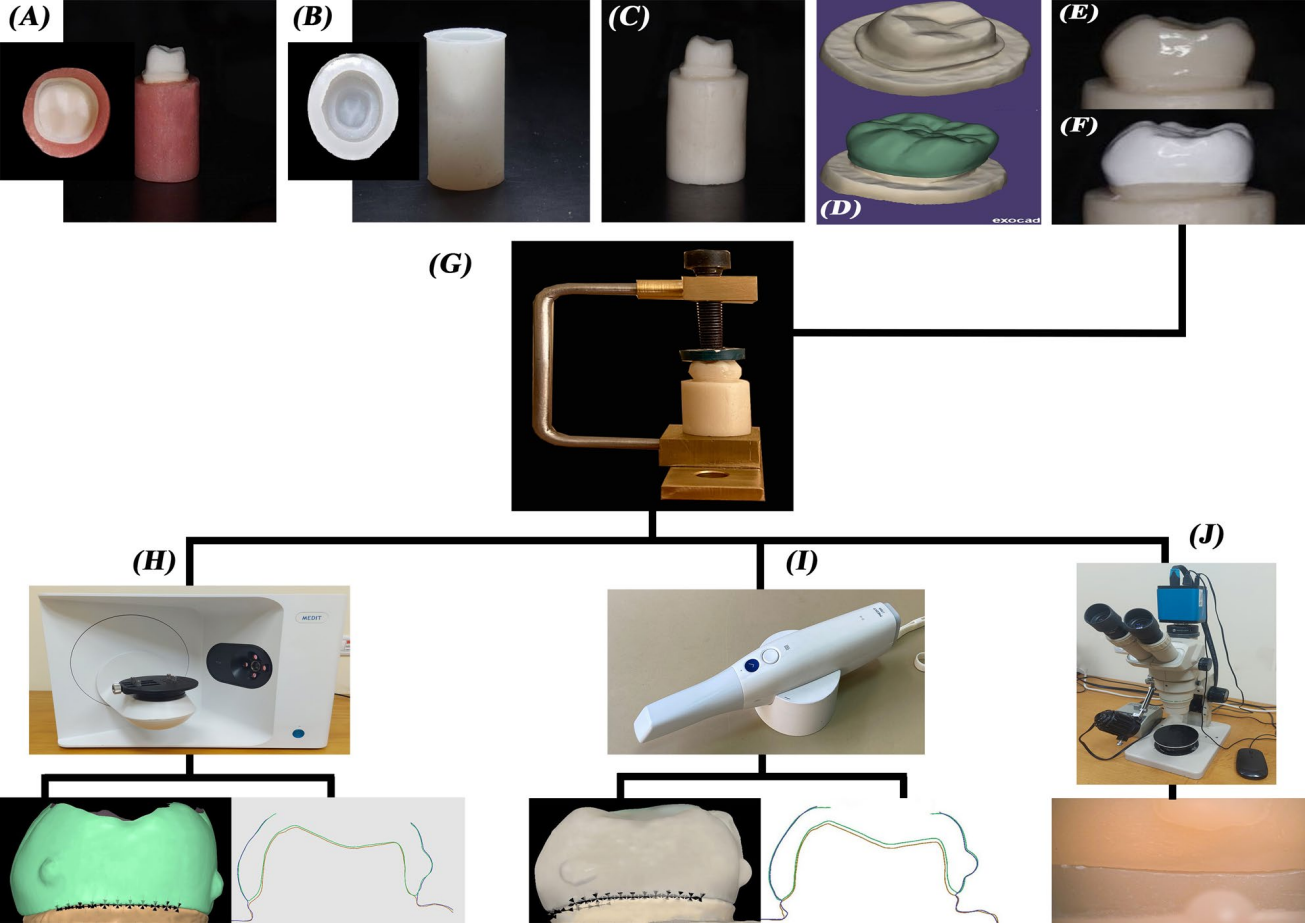
Scheme for sample preparation and assessment procedures. **A**, crown preparation on typodont. **B**, silicon mold fabrication. **C**, epoxy die fabrication. **D**, scan of the die and crown design as STL file on Exocad. **E**, milled zirconia crown. **F**, 3D printed zirconia crown. **G**, clamp used to hold specimens during scanning and stereomicroscope measurements. **H**, extraoral scanning with triple scan technique followed by marginal and internal fit assessment. **I**, intraoral scanning with triple scan technique followed by marginal and internal fit assessment. **J**, stereomicroscope assessment of marginal fit

A summary of specimen preparation and study scheme is represented in Fig. 1. A mandibular first molar typodont tooth (Basic study model, KaVo Dental GmbH, Germany) was prepared following monolithic zirconia guidelines, incorporating an occlusal reduction of 1.5 mm, an axial reduction of 1–1.2 mm with a consistent taper of approximately 8–10 degrees and a 1 mm heavy chamfer finish line [24].

**Fig. 2:**
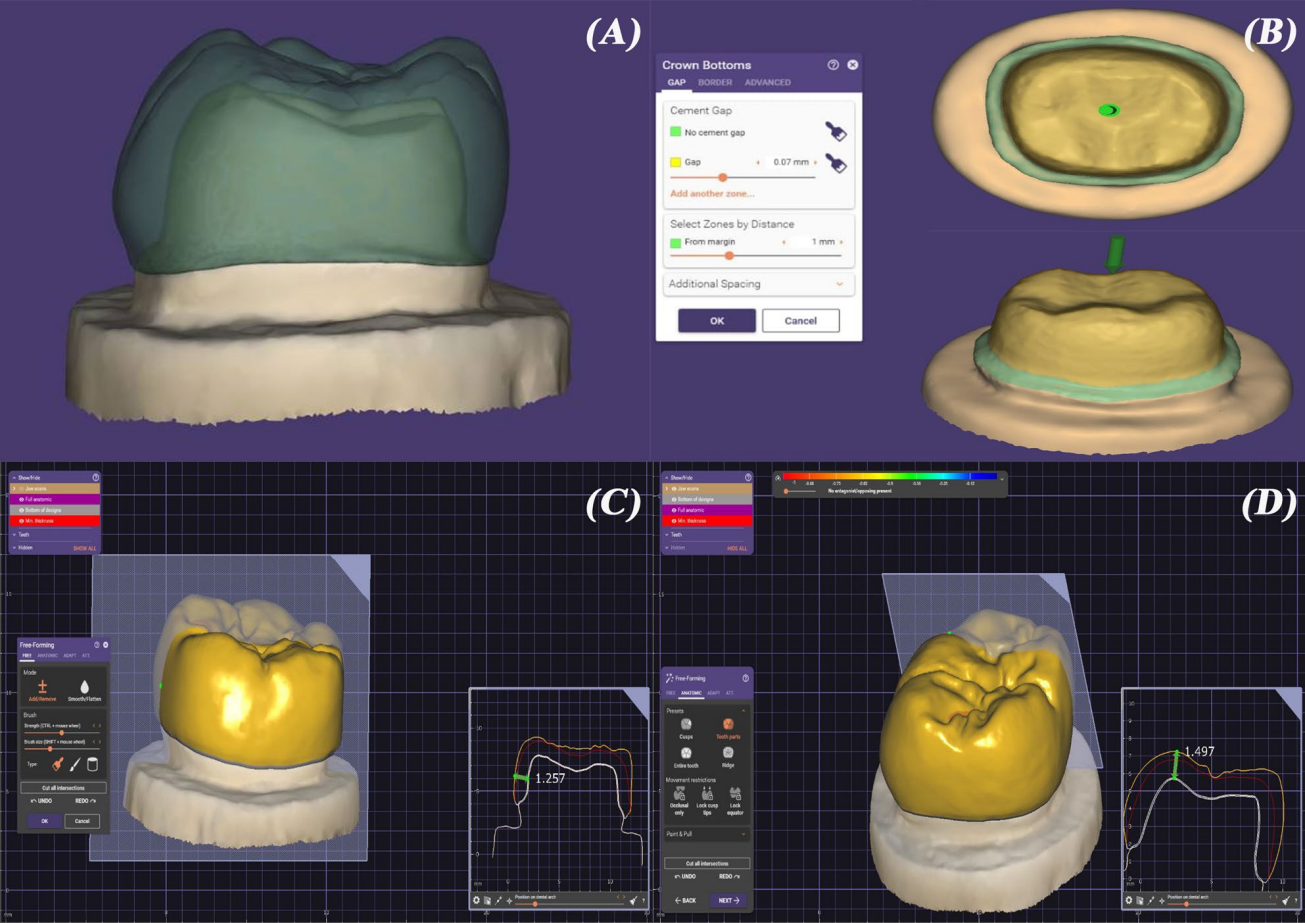
Crown design on Exocad software. **A**, die scan and crown design. **B**, cement space set at 70 μm. **C**, axial crown thickness. **D**, occlusal crown thickness

Twenty-four negative molds of the prepared typodont tooth were created using a duplicating addition silicone material (Elite Transparent, Zhermack, Italy). Each mold was filled with a dimensionally stable epoxy resin (Kemapoxy 150, CMB, Egypt), which has an elastic modulus comparable to dentin, resulting in twenty-four replicas of the prepared tooth. The definitive epoxy resin dies were randomly distributed into two groups and then individually scanned with a laboratory scanner (Medit T710, Medit Corp., South Korea). A CAD software program (Exocad version 3.0, Exocad GmbH, Germany) was used to digitally design full-contour identical crowns that mimicked lower first molars with a cement space of 70 μm started 1 mm away from the finish line [25] (Fig. 2). Once the design was finalized, the additive manufacturing (AM) (STL) files were sent for manufacturing.

For the milled group (*n* = 12), the STL files were transferred to a 5-axis milling machine (CORiTEC 150i, imes-icore GmbH, Germany) for subtractive manufacturing of the crowns utilizing milling discs made from 3 mol% yttria-stabilized zirconia (Nacera Zirconia, Dental Direkt, Germany). This was followed by sintering in a zirconia furnace (TABEO-1/M/ZIRKON-100, Mihm-vogt GmbH, Germany) in accordance with the manufacturer’s instructions.

**Fig. 3:**
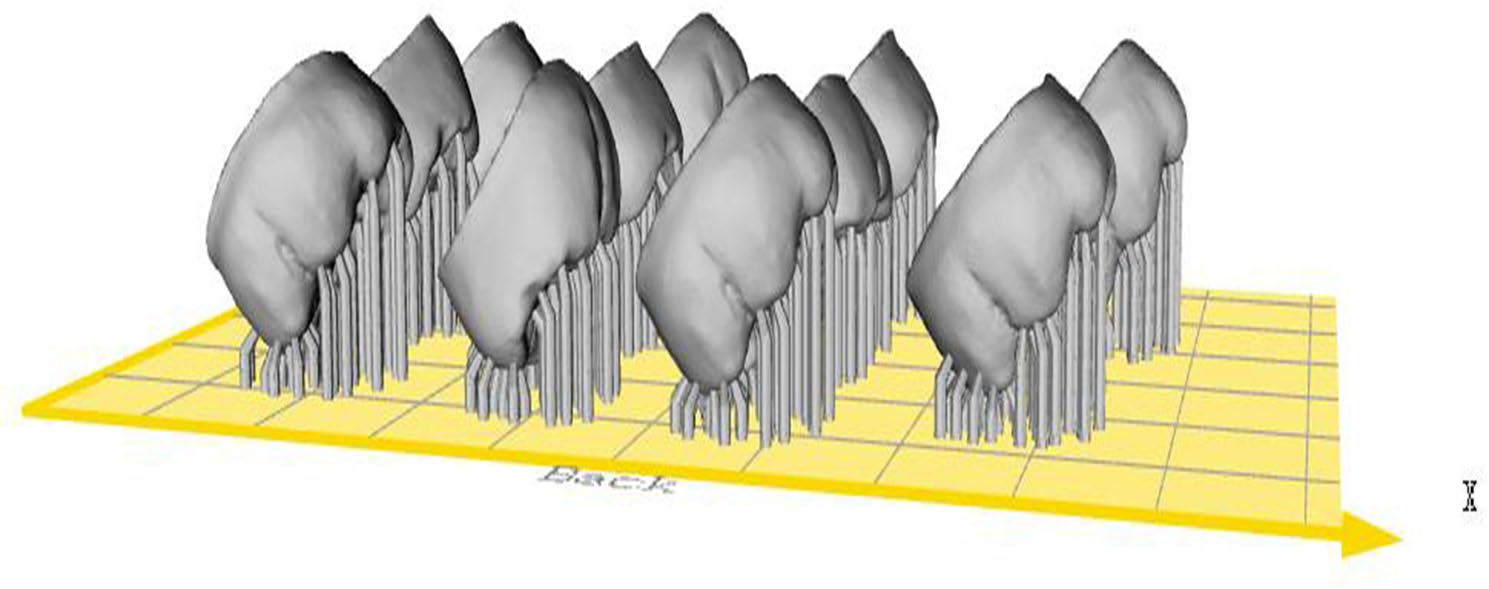
45° printing orientation of monolithic zirconia crowns

For the 3D printed group (*n* = 12), zirconia crowns were additively manufactured using a CeraFab system S65 medical printer (Lithoz GmbH, Austria) employing DLP technique. This printer selectively light cures a photosensitive ceramic slurry (LithaCon 210 3y, Lithoz GmbH, Austria) layer by layer with a printing layer thickness of 25 μm and 45° printing orientation (Fig. 3). After printing, the objects were cleaned with a designated solvent (LithaSol 30, Lithoz GmbH, Austria), the resin binder was removed, and then they were sintered to achieve fully dense zirconia crowns. Using a sintering furnace (LHTC 08/16, Naberthem GmbH, Germany), the zirconia crowns were debinded and sintered in one step.

The specimens were stored in a dry, dust free environment at room temperature to prevent any material deformation or moisture absorption that could affect the scanning accuracy.

### Triple scan method

All crowns were then individually seated without cement on their corresponding die for fit accuracy assessment. A specially designed clamp had to be fabricated to hold each crown on its die during the scanning procedure. In addition, pre-determined composite reference points had to be placed on the buccal and lingual surfaces of the crowns to help in achieving approximate alignment of the scans. The triple scan method was used to examine the marginal gap and internal fit of all zirconia specimens and was done by only one experienced operator to reduce variability in scanning technique. Scanning of each sample was performed in 3 steps: scanning of the crown on the die, scanning of the die, and scanning of the intaglio surface of the crown. The triple scan of each specimen was obtained using intraoral (Medit i700, Medit Corp., South Korea) and extraoral (Medit T710, Medit Corp., South Korea) scanning devices for comparison The resulting triple scan files of each specimen were superimposed and analyzed using a 3D analysis software program (Medit Design v.2.1.4, Medit Corp., South Korea) [26, 27].

### Internal fit measurements

**Fig. 4:**
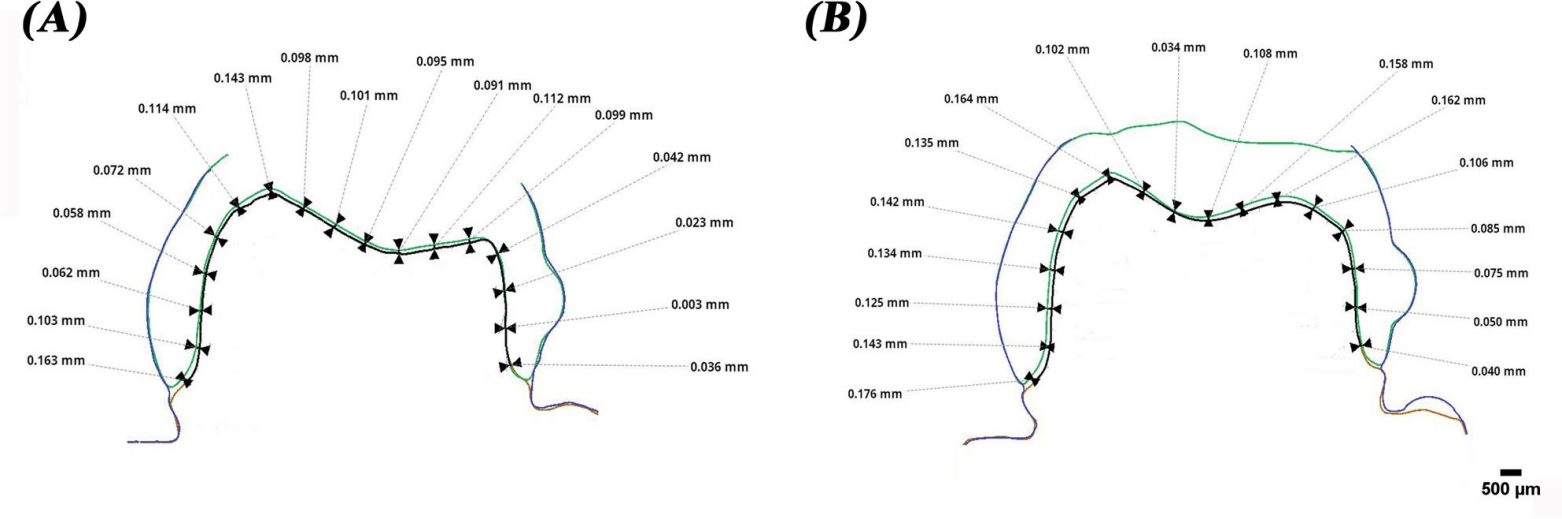
Superimposition images of scans for internal fit evaluation with measurements for (**A**), extraoral scanner images and (**B**), intraoral scanner images

The internal fit was measured by obtaining a buccolingual and a mesiodistal section of each crown after superimposition. Starting at a point 1 mm occlusal to the crown margin, the cement space was measured every 1 mm using Medit software and an average of the points obtained for each section was calculated [19] (Fig. 4). The internal fit of all zirconia crowns assessed by intraoral and extraoral triple scan protocol were compared to the 70 μm cement spacer incorporated during the designing procedure [28–30].

### Marginal gap measurements

**Fig. 5:**
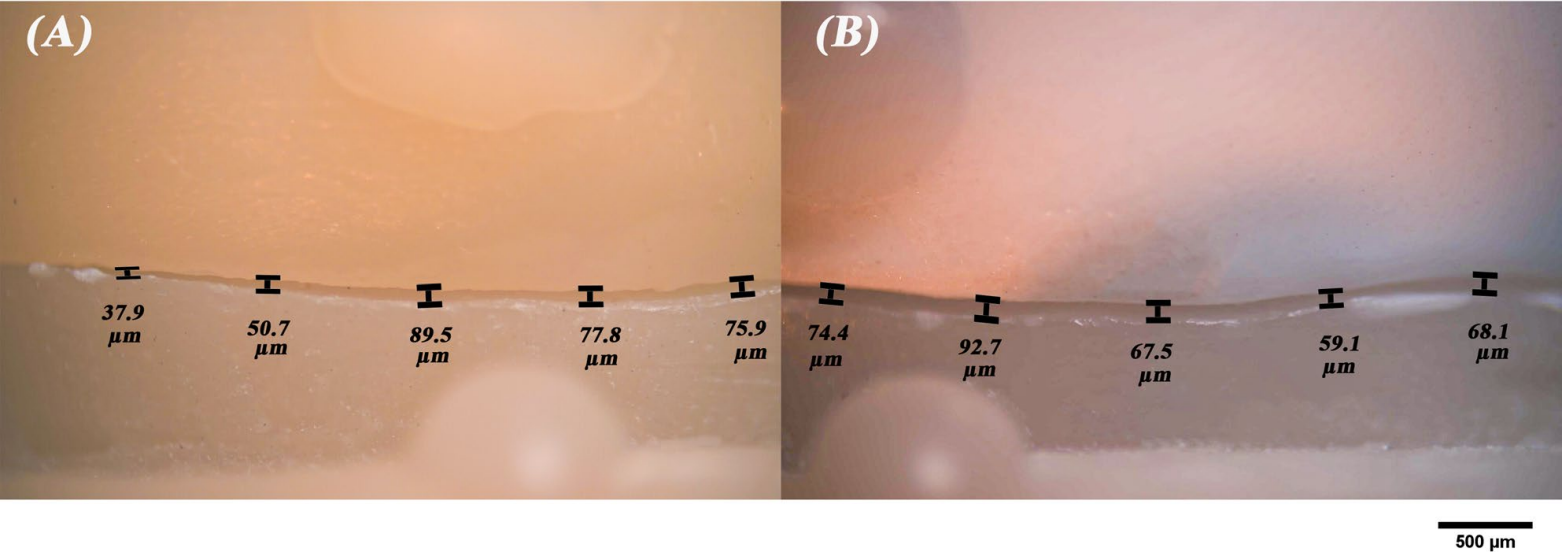
Stereomicroscope images (original magnification x50) for marginal fit evaluation of (**A**), milled zirconia and (**B**), 3D-printed zirconia

**Fig. 6:**
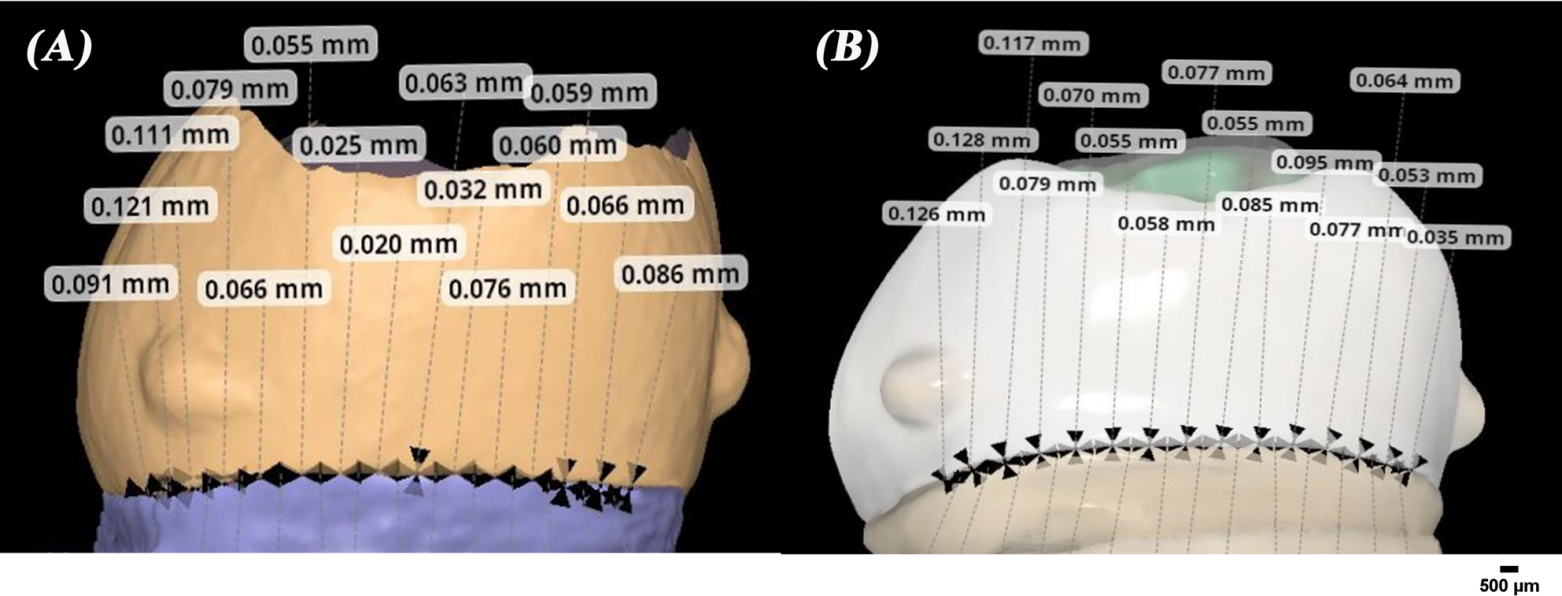
Superimposition images of scans for marginal fit evaluation with measurements for (**A**), extraoral scanner images and (**B**), intraoral scanner images

The vertical marginal gap of all specimens was measured via the triple scan protocol according to the criteria established by Holmes et al. [13]. To standardize the measurements, each axial wall was divided into three equal sections, with five measurements taken in each section. This resulted in 15 measurements for each axial wall with a total of 60 points for each specimen, which were then averaged to produce a single marginal gap value for each specimen [23]. The marginal gap of all specimens was also assessed using a stereomicroscope (B061, Olympus, Japan) with a digital camera at a magnification of x50 using the same measurement technique (Fig. 5). Then, the marginal gaps assessed by intraoral and extraoral triple scan protocol were compared to the gaps obtained by the stereomicroscope (Fig. 6).

### Statistical analysis

Normality was checked using Shapiro-Wilk test and Q-Q plots. All data showed normal distribution, so means and standard deviation (SD) were calculated, and parametric tests were used. Comparisons between the different scanners were performed using independent samples t-test (for internal fit), and one-way ANOVA followed by multiple pairwise comparisons using Bonferroni correction (for marginal fit). Comparisons of internal fit measured by different scanners with the reference standard (70 μm) were performed using one-sample t-test, with calculation of mean differences and 95% confidence intervals (CIs). Two-way ANOVA was performed to assess the effect of group and technique on internal gaps. Adjusted means, standard error (SE), and 95% confidence intervals (CI)s were calculated. Significance was set at *P*-value < 0.05. Data was analyzed using IBM SPSS for Windows (Version 26.0, IBM Corp., USA).

## Results

Table 1 shows the mean values for internal fit measurements for milled and printed zirconia measured from extraoral and intraoral scans. Both milled and printed zirconia crowns showed lower internal gap values when measured using extraoral scans (79.77 ± 4.43 μm, 82.13 ± 8.00 μm) compared to those obtained by intraoral scans (105.44 ± 2.42 μm, 107.38 ± 3.62 μm). The difference between the measurements obtained with the extraoral scanner and the intraoral one was significant for both milled and printed groups (*P* < 0.001).

**Table 1:** Comparison of internal fit of milled and 3D printed crowns for different techniques

Material	Extraoral scanner	Intraoral scanner	*P*-value
	Mean ± SD		
Milled	79.77 ± 4.43	105.44 ± 2.42	< 0.001*
3D printed	82.13 ± 8.00	107.38 ± 3.62	< 0.001*

Independent samples t-test was used

*SD* Standard Deviation

*Statistically significant at *P*-value < 0.05

When the internal fit mean values were compared to the reference standard cement gap of 70 μm, all the measurements obtained were significantly different (*P* < 0.001) compared to the standard for both study groups whether the measurements were obtained through an extraoral or an intraoral scan (Table 2).

**Table 2:** Comparison between different techniques of measuring internal fit with the reference standard (70 μm)

Material	Technique	Mean Difference from reference	95% CI	*P*-value
Milled	**Extraoral scanner**	9.77	6.96, 12.58	< 0.001*
	**Intraoral scanner**	35.44	33.91, 36.98	< 0.001*
3D printed	**Extraoral scanner**	12.13	7.05, 17.22	< 0.001*
	**Intraoral scanner**	37.38	35.08, 39.67	< 0.001*

One-sample t-test was used

*CI* Confidence Interval

*Statistically significant at *P*-value < 0.05

Table 3 shows the influence of crown fabrication technique and scanner type on the internal gap. The scanner type showed a significant effect on measurements (*P* < 0.001), while the fabrication technique had a non-significant effect (*P* = 0.15). however, the interaction of fabrication technique and scanner type was significant at *P* < 0.001.

**Table 3:** Two-way ANOVA for the effect of group and scanner type on internal gap

		Mean (SE)	95% CI	*P* value
Group	**Milled**	92.60 (1.03)	90.52, 94.69	0.15
	**Printed**	94.75 (1.03)	92.67, 6.84	
Scanner	**Extraoral**	80.95 (1.03)	78.87, 83.03	< 0.001*
	**Intraoral**	104.33 (1.03)	104.33, 108.49	

Model F: 101.83, p value: <0.001*, Adjusted R^2^: 0.87

*SE* Standard Error, *CI* Confidence Interval

P value of interaction: <0.001*

*Statistically significant at *P*-value < 0.05

The mean vertical gap values are shown in Table 4. While the extraoral scanner showed comparable marginal results with no significant difference to those obtained with the stereomicroscope regardless of the fabrication technique, the intraoral scanner showed larger gap values with a statistically significant difference to those obtained with the stereomicroscope. There was a significant difference between the intraoral and extraoral scanner used for the milled group (*P* = 0.04) as well as the printed one (*P* < 0.001) (Table 5).

**Table 4:** One-way ANOVA of marginal fit of milled and 3D printed crowns for different techniques

Material	Extraoral scanner	Intraoral scanner	Stereomicroscope	*P*-value
	Mean ± SD			
Milled	82.26 ± 16.25 **a**	93.09 ± 9.23 **b**	82.57 ± 5.25 **a**	0.04*
3D printed	86.03 ± 5.06 **a**	99.86 ± 5.30 **b**	85.57 ± 5.45 **a**	< 0.001*

a, b: Different letters denote significant differences between groups using Bonferroni correction

*SD* Standard Deviation

*Statistically significant at *P*-value < 0.05

**Table 5:** Post-hoc comparisons of marginal fit of milled and 3D printed for different techniques

Material	Technique	Compared to	*P*-value
Milled	**Extraoral scanner**	**Intraoral scanner**	0.04*
		**Stereomicroscope**	1.00
	**Intraoral scanner**	**Stereomicroscope**	0.04*
3D printed	**Extraoral scanner**	**Intraoral scanner**	< 0.001*
		**Stereomicroscope**	1.00
	**Intraoral scanner**	**Stereomicroscope**	< 0.001*

*Statistically significant using Bonferroni correction

## Discussion

In this study, the internal and marginal fit of all zirconia specimens were assessed using extraoral and intraoral scanners following the triple scan protocol. The null hypothesis was rejected as there was a significant difference between the scanners used in the assessment.

Svanborg et al. compared the triple scan protocol with the SRT technique in measuring the fit of three-unit tooth supported fixed dental prostheses and concluded that both techniques may be used for fit measurements, although it might be limited in capturing the absolute marginal gap accurately depending on the type of scanner used [16].

To ensure the accuracy of the fit assessment of the crowns on their respective dies, it was essential to evaluate both internal and marginal fit without cementing the crowns in place. To address this issue, a specially designed clamp that securely held each crown on the die, had to be specially designed to prevent any movement during the scanning process and allow the scanning procedure to proceed smoothly without interference (Fig. 1G). This setup minimized the potential for errors caused by crown displacement, which is critical for obtaining precise measurements. Additionally, to facilitate accurate superimposition analysis, composite points had to be placed at the line angles of the crowns. This process enhanced the reliability of the subsequent fit analysis using the software.

For the internal fit assessment, the SRT technique could not be used as the intaglio surfaces of the 3D printed zirconia crowns were rough causing the silicon impression to be torn upon removal. Therefore, it was decided to compare the internal fit values with the standard 70 μm cement gap that was set on the ExoCad software (Table 2).

Studies demonstrated that extraoral scanners generally outperform intraoral scanners in terms of accuracy of complex dental structures. They generally provide higher precision and accuracy in capturing surface details, which is critical for fit assessment in dental restorations, as the procedure is done in a controlled environment which reduces the variables that might affect the scan accuracy [31, 32]. Additionally, the scanning technique of extraoral scanners allows better stability of the object on the scanner table which rotates to improve the scanning angle and captures the image using multiple cameras [33].

On the other hand, an overestimate of gaps is usually associated with intraoral scans due to their limited field of view and small generated frames that require stitching and image processing [34]. Surface smoothening and noise reduction algorithms as well as post-scan processing could lead to interpolation of missing data [35]. These conditions are likely to cause distortion and discrepancies based on the technique of stitching and the algorithm used. A phenomenon that is associated with digital scanners, especially intraoral ones, is known as subsurface scattering. Light falling on the surface of the scanned object is scattered and comes out in different directions and angles instead of being reflected off the surface which leads to inaccuracy of the measurements obtained from these scans [22].

Since our work is an in-vitro study, variables such as saliva, blood, gingiva, dental restorations, and patient movement that could affect the accuracy of the intraoral scanner are excluded. However, other factors such as operator’s skill, scanning strategy, ambient light, and analysis software still have an impact on the accuracy of the obtained scan [21]. It is also recommended to use the same software of the scanners used for better accuracy, which we applied in this study by using both scanners as well as the superimposition and processing software that are provided by the same manufacturer to decrease sources of error caused by data transfer, especially between different software [36].

In the present study, 3D printed zirconia showed higher values for internal and marginal gaps regardless of the type of scanner used, which highlights the impact of the fabrication technique on the adaptation of the restoration on the die. These results were similar to those obtained by Elsayed et al. and Refaie et al. who attributed the decreased accuracy of measurement in the printed group compared to the milled one to the shrinkage that results from the printing as well as the post-processing process, especially in the DLP technique [9, 23]. Another material-related factor is the green body deformation which occurs to zirconia in its pre-sintered state. Dimensional inaccuracy and microcracks originate in that state as the partially compacted structure is highly sensitive to handling, machining, and drying stresses. These defects can be improved post-sintering, yet some might propagate in the finally sintered zirconia leading to chipping, marginal defects as well as increased gap measurements [37, 38].

The stacking of layers and the stepping effect generated due to printing also result in surface irregularities that could affect the seating of the crown on its die compared to the smooth surface achieved through the milling technique [39]. These surface irregularities also influence the reflection of light from the surface leading to diffuse reflection and scattering of light which hinders their capture by the scanner sensor and distorts the produced scan especially with intraoral scanners [22].

Despite the higher discrepancy in results of the printed specimens compared to the milled ones, the internal and marginal gaps of the printed group in this study fall within the clinically acceptable range of less than or equal 200 μm and less than or equal 120 μm respectively [40]. The use of a printing layer thickness of 25 μm in our study could be one of the contributing factors to having acceptable values of internal and marginal gaps for the printed group. The small layer thickness allowed better curing of each layer and improved surface finish thus improving crown adaptation [41]. It might have also impacted the image acquisition by the scanner as surfaces with less irregularities, such as milled surfaces, are more accurately scanned compared to rough irregular ones, such as 3D printed surfaces, as rough surfaces influence the reflection and scattering of light from object to scanner [42].

Marginal and internal gaps lead to cement dissolution, microleakage, secondary caries, periodontal inflammation, and affect the overall longevity of zirconia restorations. The assessment done in this study would be of aid to clinicians in deciding which scanning method would be more reliable in measuring gaps and ensuring the fit of fixed restorations.

Limitations of this study include using only one type of extraoral and intraoral scanner and conducting the study on single crowns. The current study was also limited by its focus on just one type of 3D printed zirconia material. Additionally, the workflow has not been tested under clinical conditions as the presence of blood and saliva as well as patient’s interaction could influence the results. Future research should explore a variety of 3D printed zirconia materials produced with different 3D printing technologies to enhance the understanding of their performance. More studies should be conducted to detect the marginal and internal fit using different types of extraoral and intraoral scanners with long span fixed dental prostheses.

## Conclusions

The choice of scanner type as well as the fabrication technique play a key role in influencing accuracy of zirconia restorations. Within the limitations of this study and based on the results obtained from the two scanners used, gaps were more accurately depicted by the Medit T710 extraoral scanner measurements compared to Medit i700 intraoral scanner measurements. Although 3D printed zirconia showed higher discrepancy in internal and marginal gaps, both milled and 3D printed zirconia expressed internal and marginal fit that is within the clinically acceptable range.

## Data Availability

The data that support the findings of this study are available from the corresponding author upon reasonable request.

## Abbreviations

3D: three dimensional
AM: additive manufacturing
SLS: selective laser sintering
FDM: fused deposition modeling
SLA: stereolithography
DLP: direct light processing
SRT: silicone replica technique
CAD: computer-aided design
STL: standard tessellation language
SD: standard deviation
